# Self-Erasable Nanocone Antireflection Films Based on the Shape Memory Effect of Polyvinyl Alcohol (PVA) Polymers

**DOI:** 10.3390/polym10070756

**Published:** 2018-07-09

**Authors:** Peng Li, Yu Han, Wenxin Wang, Xinlong Chen, Peng Jin, Shengchun Liu

**Affiliations:** 1 Heilongjiang Provincial Key Laboratory of Metamaterials Physics and Device, Heilongjiang University, Harbin 150080, China; lipenghit@126.com (P.L.); cxl980253425@163.com (X.C.); 2 Acoustic Science and Technology Laboratory, College of Underwater Acoustic Engineering, Harbin Engineering University, Harbin 150001, China; 3 Research Center of Ultra-Precision Optoelectronic Instrumentation, Harbin Institute of Technology, Harbin 150080, China; 1991hanyu@163.com (Y.H.); P.Jin@hit.edu.cn (P.J.); 4 State Key Laboratory of Marine Resource Utilization in South China Sea, Hainan University, Haikou 570228, China; wangwenxin.999@163.com

**Keywords:** self-erasable, antireflection film, shape memory polymer

## Abstract

Arrays of nanostructure that are capable of broadband antireflection and light trapping properties are implemented in photovoltaic and photonic devices. However, most of the existing antireflection films have been hindered by a complicated fabricated method and structurally rigid. Here, we report a simple preparation method for self-erasable nanocone antireflection films using the surface replication method. Arrays of nanocone with sub-100 nm surface features could be replicated easily on the shape memory polyvinyl alcohol (PVA) film, and are erased by thermal stimulation. The reflectivity of self-erasable antireflection film can be switched from the 4.5% to 0.6% averaged over the visible spectral range by controlling the temperature below or above 80 °C. Theoretical simulations have been demonstrated. The unique smart film is expected to be used to further extend the application of smart optical windows and digital screens.

## 1. Introduction

When light travels at the abrupt interface with different refractive index, it occurs the reflection and refraction phenomenon, accompanied with light energy being redistributed. In many applications, the strong reflected light and the optical light loss are undesired. For example, the glare of reflected light leads to vision impairment. Optical loss of the light reflection reduces the efficiency of photovoltaic devices and influences on the imaging quality. Various anti-reflection films have been developed to mitigate the strong reflected light and optical light loss. Conventionally, single/multilayer antireflection films are employed in order to suppress the optical reflection based on light destructive interference effect [1,2]. However, single/multilayer antireflection films are limited by a narrow range of operating wavelengths, incident angles, multi-materials performance mismatch, costly fabricated methods, and difficult assembly precision.

Meanwhile, arrays nanostructures have attracted significant attention because of their unique advantages, such as broadband antireflection, flexible light trapping property, high surface area, and easy forming. A lot of properly arrays nanostructures have been designed and fabricated, such as nanodomes [3], nanospheres [4,5], nanocones [6,7], nanowires [8], nanopillars [9], and nanopyramids [10]. Various researches have been reported on patterning nanostructures using the metals, glass, semiconductor, and polymers materials substrates for effectively reducing optical reflection [11,12,13,14]. Optical polymer materials as substrate have attracted more attention due to their flexible optical properties, low cost, and easy processing. Li J. et al. [15] prepared an antireflection polymethylmethacrylate (PMMA) film with nanonipples arrays by template-based nanoimprinting technology, which exhibits an excellent antireflection effect. Average reflectance can reduce to 0.6% over the visible spectral range. Tsui K. H. et al. [16] reported an additional antireflection polydimethylsiloxane (PDMS) film with nanocone arrays that could be attached to Cds/CdTe solar cells surface for efficient utilization of the incident photons. Zhang W. et al. [17] fabricated a polycarbonate (PC) nanocone films with antireflection and condensate micro-drop self-removal function. The polymer films with nanostructures promote the development of antireflection films and demonstrate appealing performance, but they are still hard to realize the versatility and smart switching mechanism, which are crucial for their flexible performance optimization.

The optical modulation of the antireflection films is conventionally induced by nanostructure changes. Many functional nanostructures have been proved to be constructed using smart materials. Hiller J. et al. [18] explored an intelligent multilayer polymer film with a nanoporous structure that could be erased, which endowed the film with reversible antireflection properties. Alternatively, shape memory polymers (SMPs) are promising candidates to achieve the light modulation, because of their mechanical response when triggered by an external stimulus, such as temperature, electricity, light, and ionic concentration [19,20,21]. SMPs have been extensively investigated in various areas, such as actuators [22], sensors [23], textiles [24], and micro optics [25,26,27]. An optically transparent polyvinyl alcohol (PVA)-SMP was used in this work, which is low cost commodity polymer and exhibits high transparency, adjustable mechanical property, fast response speed, easy processing, and driving. These unique mechanical and optical properties make it fit for the self-erasing antireflection film applications.

In this study, the self-erasable nanocone antireflection films that are based on the shape memory effect of PVA polymers were fabricated by precise controlling the hot embossing process. The nanocone arrays with fixing identical heights and periods can be easily reprinted in the flat PVA-SMP film. Reprinted nanocone arrays can effectively depressing reflection and keeping a durable station at the room temperature. Due to their micro-shape memory effect, the nanocone structures can be easily disappeared through high temperature stimulation. Intriguingly, it enables a new route to fabricate a flexible self-erase antireflection film, which can considerably improve the smart film development.

## 2. Materials and Methods

### 2.1. Materials and Preformed PVA-SMP Film Sample

All of the materials of the nanocone antireflection films are readily available. Polyvinyl alcohol (PVA, >99% hydrolyzed, average molecular weight 85,000–124,000) and Glutaraldehyde (GA, ~50% in water) were purchased from Sigma Aldrich (Darmstadt, Germany). All of the chemicals were used as received without further purification. Deionized water with the conductivity less than 2 µs/cm was used throughout the experiments.

**Figure 1:**
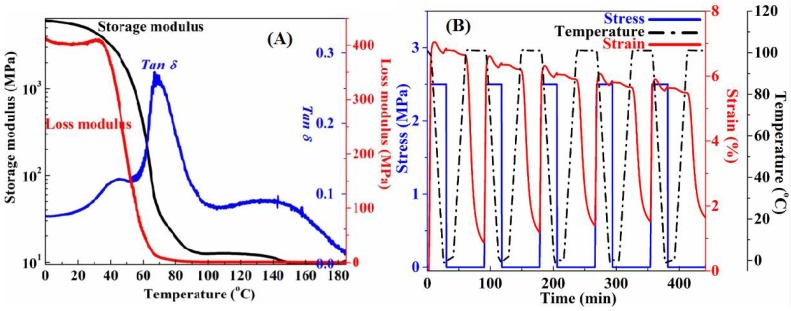
(**A**) Dynamic mechanical analysis (DMA) curves of the polyvinyl alcohol-shape memory polymers (PVA-SMP) sample, including Storage and Loss modulus as a function of temperature, and Tanδ as a function of temperature. (**B**) Stress-controlled cyclic thermo-mechanical testing curves of the PVA based shape memory polymer sample.

8 g PVA and 100 mL deionized water were added into a glass bottle with a thermometer and a mechanical stirrer. The mixtures were stirred for 1 h at the room temperature to the PVA particles full swelling. Then, the mixtures were heated to 98 °C for 3 h, continuously stirred until PVA dissolved completely. The PH value of the solution was adjusted to 3.5 and then 3 mL GA was added in the solution. After stirring for 2 h, the bubble-free PVA precursor sol was obtained in vacuum and then poured into the mold at the room temperature. It was dried for 48 h at room temperature, and then for 3 h at 60 °C and 24 h at 50 °C in vacuum oven to obtain the preformed flat PVA-SMP film sample. The PVA-SMP has a glass transition temperature (*T*_g_) of 68 °C, and a glass transition zone of 60–100 °C, as determined from the Dynamic Mechanical Analysis (DMA) measurements (in Figure 1A). In terms of the operation cycle, the PVA-SMP that is reported here is a one-way memorizing material. When the SMP sample with the temporary shape recovers its original shape above the *T*_g_, one shape memory cycle was completed. The SMP sample can be re-deformed by heating and embossing process. However, a SMP sample can be used repeatedly from predesign nanocones surface to fully smooth surface and be convenient for a wide range of application requirements. To illustrate the shape memory cycle times, five shape memory cycles were performed using DMA Q800/RSA3 (TA Instruments, New Castle, DE, USA). The cyclic thermo-mechanical testing curves of the PVA-SMP sample are shown in Figure 1B. The sample exhibited good mechanical properties, the fixity and recovery ratio of the samples are all above 90%. Therefore, the material excellent shape memory effect and cyclic fatigue resistance can be used as the dynamic tunable optical components.

### 2.2. Fabrication of Self-Erase Nanocones Films

The aluminum (Al) template with inverse nanocone arrays used as the nanoimprinting mold was prepared by the two-step anodization process. An electrochemically polished clean, Al foil was initially anodized under appropriate conditions. An ordered hole arrangement at the bottom of the alumina were generated after a long period. Thereafter, the inverse nanocone array was fabricated by a multi-step anodization and wet etching process on the imprinted Al foil in an acidic solution with a proper direct-current (DC) voltage [28,29]. Afterwards, a layer of fluoroalkylsilane solution (diluted with ethanol) was coated on the surface of the obtained alumina template as an anti-sticking layer between the inverse nanocone array and PVA for easy peeling off of patterned PVA film from the template, subsequently. The Al template was cleaned using piranha solution before use. The PVA flat film of 20 mm×20 mm ×0.5 mm was reprinted the nanocone arrays using Al template with an identical pitch (Λ=100 nm) and height (*H* = 200 nm) by relatively high pressure (50 bar) and temperature (90 °C). Afterwards, a PVA nanocone film with the same diameters of inverse template (the base and top of the unit cone are 90 and 40 nm) was obtained by directly peeling off PVA film from the Al template.

### 2.3. Characterization

Dynamic mechanical analysis (DMA) was carried out on DMA/SDTA861e (Mettler-Toledo, Greifensee, Switzerland) and DMA Q800/RSA3 (TA Instruments, New Castle, DE, USA) in a tension mold. The pre-designed nanocone arrays images of the PVA films surface were obtained by field emission environmental scanning electron microscopy (SEM, Quanta 200 FEG, FEI, Hillsborough, OR, USA), Atomic Force Microscope (AFM, Dimension Icon, Bruker, Karlsruhe, Germany), and optical microscopy (VHX-900, Keyence, Osaka, Japan). The transmission spectra of the SMP films were obtained using a UV–VIS spectrophotometer (Shimadzu UV-3600, Shimadzu, Kyoto, Japan). The reflectance of the SMP films with and without nanocone structures was recorded by a home-built reflectance measurement set up.

## 3. Results and Discussion

### 3.1. Characterization of PVA-SMP Film with Nanocone Arrays

**Figure 2:**
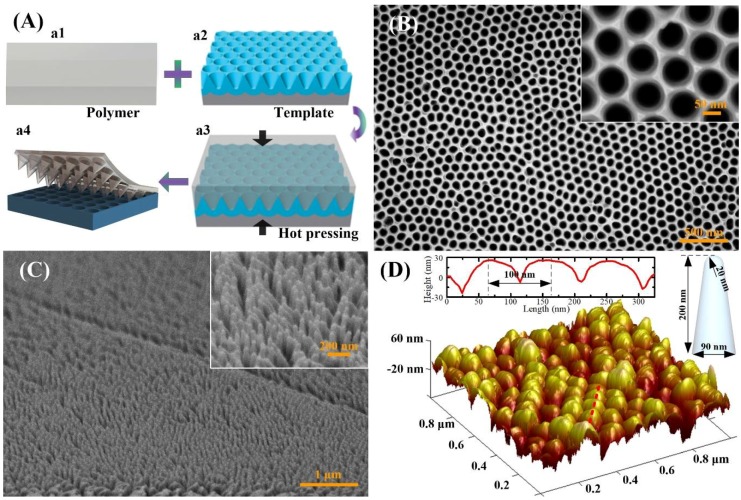
(**A**) Scheme of the fabrication processes for nanocone PVA film. (**a1**) the prepared PVA film with certain thickness; (**a2**) Al template with inverse nanocone arrays used as thermal embossing nanoimprint mold; (**a3**) A performed flat PVA film is hot embossed into the Al template; (**a4**) Carefully peeling off the nanocone patterned PVA film from the template. (**B**) The SEM image of the Al template and the scanning electron microscopy (SEM) (**C**) and Atomic Force Microscope (AFM) (**D**) images of the nanocone patterned surface of the PVA film, showing that the nanocone with height of ~200 nm, and pitch of ~100 nm. Inset in (**D**) shows the shape of an element of the nanocone structure patterned on the PVA film.

Figure 2A shows the schematics of the fabrication process. The flat PVA-SMP film can be re-printed with high resolution and precision. The PVA-SMP film is squeezed into the inverse nanocone arrays of Al template under relatively high pressure and above the glass transition temperature (*T*_g_). A replica is then created after the template separation at the room temperature. The template without destroying can be easily peeled off, and the film can be reused multiple times. Figure 2B shows SEM micrographs of the inverse nanocone structures of the Al template, honeycomb cavities can be easily observed with diameter of ~90 nm and pitch of ~100 nm. Figure 2C,D show the SEM and AFM morphologies of the replicated nanocone arrays on the PVA-SMP film, showing that the cones on PVA-SMP are rounded tip cones with a height of 200 nm. The cladding diameter is about 90 nm and the tip width is about 40 nm. The clear and precise boundary guaranteed the reproducibility of the nanostructures with sub-100 nm. To further demonstrate the reliability of the fabrication method, the AFM image detailed the cross-sectional nanocone with periodicity of 100 nm. We note that the AFM tip cannot detect the deep bottom of the nanocone array due to the high aspect ratio of nanostructures. Inset in Figure 2D demonstrates the geometry of the rounded tip cone that was replicated by the PVA-SMP film. Notably, other nanostructures surfaces also can be reprinted with similar approach to apply for different applications areas. Large-scale nanocone arrays can be conveniently obtained through a separate thermal embossing process.

### 3.2. Theoretical Model

**Figure 3:**
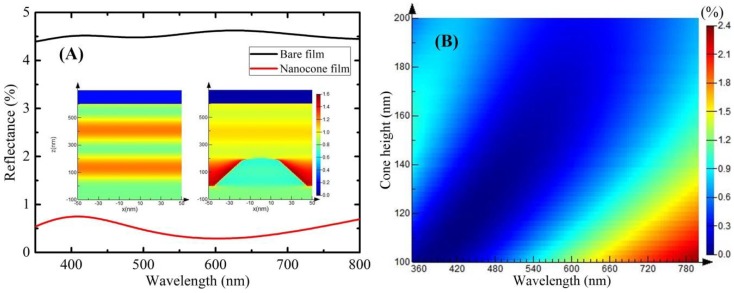
(**A**) Finite-difference-time-domain (FDTD) simulations of reflectance spectra and the cross-sectional electric field intensity distribution of electromagnetic wave at 550 nm wavelength; and, (**B**) Simulated integrated reflectance at different cone heights.

For identifying and optimizing the antireflection properties of the nanocone film, the optical simulations of the bare film and nanocone film were performed by the Finite-difference-time-domain (FDTD) simulations. In the simulation, the refractive index of the PVA film is 1.54, which is measured using an ellipsometer. The wavelength of electromagnetic (EM) wave is 550 nm. FDTD simulations only perform the one-side (air/medium interface) reflection features of the films. The theoretical reflectance curves of bare/nanocone PVA-SMP films are obtained using the Lumerical FDTD Solutions analysis software. Figure 3A shows the FDTD simulations of reflectance spectra for film with and without rounded tip nanocones throughout the visible light range. The bare film exhibits a high reflectance of around 4.5% in the visible region. The reflectance largely decreased to 0.5% for the film with nanocone arrays. This means that the nanopatterned PVA film can improve the antireflection property for the film with a factor of 7.5. The ${\left|E\right|}^{2}$ distribution of the EM plane wave with bare film and nanocone film are illustrated in the inset of Figure 3A. For the left inset, the top surface of the PVA film located at *Z* = 0 nm. For the right inset, the top of the nanocone locates at *Z* = 200 nm. Note that the color index at the specific location reflects the intensity of ${\left|E\right|}^{2}$ at that point, normalized with the EM plane wave propagating in free space. The stripe-like patterns indicate the interference between the incoming wave with the reflected wave. The color above *Z* = 600 nm manifests the intensity of the reflected wave. It can be seen that the existence of rounded tip nanocones results in weaker interference and weaker intensity of the reflected wave. In addition, it can conspicuously be seen from the ${\left|E\right|}^{2}$ distribution around the nanocone structure that the EM wave energy can be efficiently coupled between nanocones and the multi-reflection effect of cones will finally guide the EM wave energy into the nanocone. The simulation results show that the EM wave energy can be effectively coupled into the nanocone. It will decrease the interference effect and the intensity of the reflected wave.

The geometric factors of the nanocones, i.e., pitch and height, can be largely tuned with the established approach. But, in the nanoimprint process using a certain template, the replication accuracy is directly determined by the cone height. Therefore, systematic optical simulations have been performed in order to identify the optimal structures for light trapping. The reflectance spectra of nanocones with different heights were simulated to investigate the influence of their geometry on the antireflection effect, as shown in Figure 3B. It can be seen that when the cone height is above 170 nm, the nanocone film exhibits extremely broadband low reflectance (usually below 0.9%) crossing the entire visible range. The reflectance of nanocone with fixed height (below 150 nm) has an obviously increasing trend with the incident wavelength occurring red shift. The theory results can be developed to optimize nanostrutures design and to verify the experiment results.

### 3.3. Optical Properties Measurements of Bare Film and Nanocone Film

**Figure 4:**
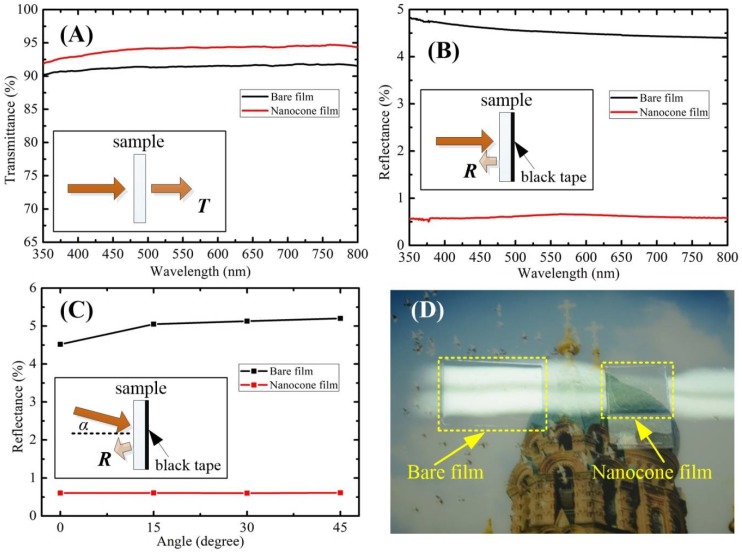
(**A**) Transmittance measurements of bare film and nanocone film; (**B**) Reflectance measurements of bare and nanocone film obtained for the light incident vertically; (**C**) Reflectance measurements of bare and nanocone film obtained for the light incident obliquely from 0° to 45°; and, (**D**) Visual effect of the bare film (on the left) and nanocone film (on the right) exposed to the strong lighting.

For electrical screen displaying and optical protection applications, the antireflection film should possess high transparency in order to ensure the clear observation and precise information transmission. In subsequent experiments, the optical properties (transmission and reflection) of the nanocone film were exploited. Transmittance measurements were performed on the bare film and nanocone film from 350 to 800 nm. As can be seen in Figure 4A, both bare film and nanocone film exhibit a relative higher transmittance (above 90%). The average transmission is increased from around 91.5% of the bare film to an average of 94.5% of the nanocone film. The nanocone existence largely improves the light transmittance of the PVA film.

To measure the air/medium interface reflection, black tapes as light trap were mounted on the back surface (i.e., medium/air interface) of films. Vertical specular reflectance of PVA films with and without nanocones was measured in the visible region, as shown in Figure 4B. The reflectance of PVA film decreased from around 4.5% of the bare film to of 0.6% of nanocone film. It is indicated that the nanocone arrays exhibit a strong light trapping ability and allow for light propagation across the interface. The experimental reflectance in Figure 3B qualitatively agrees with the simulation reflectance in Figure 3A.

One point should be noted that the transmission and reflection measurement results in Figure 4A,B were obtained by the normal incident light. In the practical application, the angle of the incident light is not usually normal and fixing. Therefore, we investigated the reflectance of the bare film and nanocone film at several angles of the incident light. The reflectance of the bare film and nanocone film at angles 0, 15, 30, and 45 degree were obtained via multi-measurement average to achieve reliable results. Five different random positions of every film sample were selected for measured in the reflectance testing experiment. The average reflectance measurement results were revealed in Figure 4C. Remarkably, there is not significant change of the nanocone film reflectance with the different incident degree. The reflectance maintains around average 0.6%. While the reflectance of the bare film increases from 4.5% to 5.3% with the incident degree increasing. Reflectivity measurements revealed that the nanocone film is strongly antireflective through the entire visible spectrum and over a wide range of incident angles (0−45°). Figure 4D shows a photograph of the bare film (left) and the nanocone antireflection film (right) exposed under strong lighting. A clear decrease in reflectance for PVA film can readily be observed by naked eyes. The strong surface specular reflection of the bare PVA film causes information losing underneath the film and brings vertigo to the viewer. However, the nanocone antireflection film greatly improves the visual comfort, and the information underneath the PVA film can also be easily detected.

### 3.4. Self-Erase Properties of PVA-SMP Film with Nanocone Arrays

**Figure 5:**
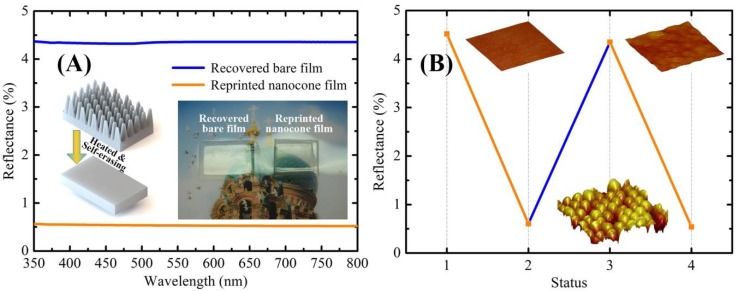
(**A**) Reflectance measurements of recovered bare PVA film and reprinted nanocone PVA film obtained for the light incident vertically (Inset figures show macroscopic optical images of the corresponding recovered bare film and reprinted nanocong film upon the color picture); and, (**B**) Reflectance of a PVA film with nanocone arrays under a shape memory cycle (Insets show the AFM images of the PVA film with three states, including original surface, reprinted nanocone arrays, and recovered surface).

Due to the shape memory effect of the PVA nanocone film, the original surface structures and the optical properties can be recovered with excellent reversibility in the antrireflection properties. To demonstrate the self-erase properties of PVA-SMP antireflection nanocone film, the reflectance change between the bare film and the nanocone film of the PVA-SMP films were qualitatively and quantitatively recorded throughout the entire thermal recovery process in Figure 5A. The flat PVA film (thickness of 50 μm) with smooth surface (original shape) was reprinted into nanocone film (compressed shape) by thermal embossing technology. The reflectance of the reprinted nanocone film was observed around 0.6%. The PVA-SMP film with reprinted nanocone shape was installed on a transparent ITO electric heating film for thermal simulation experiment. Under a continuously applied voltage (~21 V) and approximately 25 s later, the PVA-SMP film was reheated above *T*_g_, and the original shape (smooth surface) was recovered quickly. Correspondingly, the reflectance of film switched from 0.6% to around 4.4%, which is close to the reflectance of the bare film. Figure 5B shows that the reflectance changes of a PVA film in three states from left to right, which are preformed flat, reprinted nanocone, and recovered bare state) under a shape memory cycle. Inset AFM images of the PVA films (Figure 5B) reveal the nanocone arrays surface that are reformed and erased states during the shape recovered cycling process. Figure 5A observed the visual effect of the recovered bare film (on the left) and the reprinted nanocone film (on the right) exposed to the strong lighting.

## 4. Conclusions

In this paper, we propose and demonstrate a thermally activated self-erase antireflection nanocone film in both theoretical simulation and experiment based on the shape memory effect of the PVA film. The nanocone arrays can be easily reprinted on the PVA-SMP film surface for any desired display format in a short time. The reflectivity of the self-erasable antireflection film can be switched over the visible spectral range due to PVA shape memory effect. This new self-erase mechanism is explained, broadens the fundamentals of shape-memory polymers in micro-deformation, and consequently, can be exploited to develop a controllable optical antireflection film for intelligent displays.
